# Influence of an Alkaline Activator and Mineral Admixture on the Properties of Alkali-Activated Recycled Concrete Powder-Foamed Concrete

**DOI:** 10.3390/ma18112567

**Published:** 2025-05-30

**Authors:** Yongfan Gong, Chao Liu, Zhihui Zhao, Zhengguang Wu, Bangwei Wu

**Affiliations:** College of Architectural Science and Engineering, Yangzhou University, Yangzhou 225127, China; yfgong@yzu.edu.cn (Y.G.); yzuliuchao@163.com (C.L.); zgwu@yzu.edu.cn (Z.W.); wubw@yzu.edu.cn (B.W.)

**Keywords:** recycled concrete, alkali-activated, foamed concrete, porosity, thermal conductivity

## Abstract

Alkali-activated recycled concrete powder-foamed concrete (ARCP-FC) is a new type of insulation architectural material, which is prepared using recycled concrete powders (RCPs), slag powders, fly ash, and sodium silicate. In this study, the influence of the water-to-cement (W/C) ratio, the Na_2_O content, and the mineral admixture content on the mechanical strength, physical properties, and thermal conductivity of ARCP-FC were investigated. The results showed that the compressive strength and dry apparent density of ARCP-FC decreased with the increase in the W/C ratio. In contrast, the water absorption rate increased as the W/C ratio increased. Fewer capillaries were formed due to the rapid setting property, and the optimal W/C ratio was 0.45. The compressive strength and dry apparent density first decreased and then increased with the increase in Na_2_O content. Too high Na_2_O addition was not conducive to the thermal insulation of ARCP-FC, and the optimal Na_2_O content was 6%. The compressive strength and dry shrinkage gradually decreased, while the water absorption gradually increased as the fly ash content increased. Fly ash improved deformation, and the pore was closed to the sphere, reducing the shrinkage and thermal conductivity. The optimal mixture of ARCP-FC consisted of 60% recycled concrete powders, 20% slag, and 20% fly ash. The density, porosity, compressive strength, and thermal conductivity of ARCP-FC were 800 kg/m^3^, 59.1%, 4.1 MPa, and 0.1036 W/(m·K), respectively. ARCP-FC solved the contradiction between compressive strength and dry apparent density, making it a promising building material for external insulation boards and insulation layers.

## 1. Introduction

Foamed concrete, as a promising thermal insulation building material, is prepared using cementitious material through the physical foaming method. It demonstrates the advantages of being lightweight, energy-conserving, and environmentally friendly. The traditional foam concrete is dependent on cement-based materials and has limitations in terms of its high cost and increasing energy consumption [1]. However, alkali-activated foamed concrete is prepared using slag and fly ash powder and is foamed in situ with a foaming agent, showing superior performance, with an apparent density of 534~1030 kg/m^3^ and a strength of 1.8~5.1 MPa [2,3,4]. Moreover, the higher viscosity of alkali-activated foamed concrete can restore more bubbles in the paste and form a large mass of uniform closed pores, resulting in better thermal insulation performance.

Alkali activators, such as sodium silicate and NaOH, have been widely used to stimulate the activity of industrial waste [5,6,7]. Gu et al. [8] reported the influence of glass waste on the mechanical behavior of alkali-activated slag. Yin et al. [9] explored the use of spraying sodium silicate to stabilize recycled demolition aggregates. SiO_2_ and Al_2_O_3_, in recycled concrete powders (RCPs), exhibited potential reactivity and could replace slag or fly ash in the preparation of alkali-activated materials. Shi et al. [10] found that concrete wastes were promising precursors for alkali-activated material, and the wastes were divided into high-Ca and low-Ca types. It is obvious that the activators of Na_2_SO_4_, NaOH, and sodium silicate can significantly enhance the reactivity of RCPs and lead to a notable increase in strength [11,12,13].

The crucial factor that influences the strength of foam concrete is the dry apparent density [14]. The incorporation of sodium lauryl sulfate showed good stability [15]. Souza et al. [16] prepared alkali-activated foamed concrete with recycled red brick powder, using lime and gypsum as activation materials. Adding 25% CaO can significantly reduce the apparent density and improve the mechanical properties of foamed concrete. He et al. [17] found that surfactants significantly influenced the performance of foamed concrete prepared with alkali-activated fly ash, and combined activation using sodium hydroxide and lime powder resulted in increased mechanical behavior compared to the adoption of a single activator. Zhang et al. [18] used aluminum powder as the foaming agent and prepared the alkali-activated metakaolin-foamed concrete with a mechanical strength of 0.6~3.50 MPa and a dry apparent density of 250~600 kg/m^3^. Studies [19,20,21] reported fly ash and slag powders added to prepare the alkali-activated foamed concrete, with a thermal conductivity of 0.07~0.139 W/(m·K), a 28-day compressive strength of 0.6~3.50 MPa, and a dry apparent density of 250~600 kg/m^3^. The density of alkali-activated slag-fly foamed concrete was 200 kg/m^3^~1200 kg/m^3^ [22]. It is obvious that the density is a critical factor that influences the mechanical behavior and differentiation of the dry apparent density of foamed concrete, reaching a mechanical strength in the range of 0.50~44.98 MPa. Yang et al. [23] studied the alkali-activated slag foamed concrete with an apparent density of 300~500 kg/m^3^ and a thermal conductivity of 0.095~0.13 W/(m·K). Shi et al. [24] blended cenospheres with alkali-activated slag-fly foamed concrete and obtained an optimal performance with an apparent density of 405.7 kg/m^3^, a compressive strength of 3.4 MPa, and a thermal conductivity of 0.10 W/(m·K). Alnahhal et al. [25] used the fuel ash of palm oil as a substitute for fly ash to prepare alkali-activated foamed concrete, made with 20% fuel ash palm oil, which showed a compressive strength of 6.1 MPa and an apparent density that ranged from 1193 kg/m^3^ to 1344 kg/m^3^. When the samples were cured at 60 °C for 6 h, the dry apparent density of alkali-activated foamed concrete ranged between 600~650 kg/m^3^, the 28-day compressive strength was ≥3.5 MPa, along with a thermal conductivity of 0.178 W/(m·K) and a shrinkage value of 0.87 mm/m.

Previous studies have mainly focused on the composition, preparation, and performance of foamed concrete, but few studies have considered RCPs as raw materials of alkali-activated foamed concrete. Specifically, a comprehensive analysis of the cooperative effects and pore structure evolution within composite materials containing cement, ground granulated blast furnace slag, fly ash, and RCPs is still lacking. RCPs are derived from the production and processing of recycled aggregate [26], and SiO_2_ and Al_2_O_3_ in the RCPs have shown potential hydration activity [27] after mechanical grinding and chemical excitation. The partial replacement of RCPs with mineral admixture enhanced the reaction rate and mechanical behavior of alkali-activated recycled powder concrete [28]. This paper replaced slag powder and fly ash with RCPs and investigated the influence of RCP content on the mechanical properties, dry apparent density, drying shrinkage, and thermal conductivity of alkali-activated recycled concrete powder-foamed concrete (ARCP-FC). The development of ARCP-FC made with solid waste is beneficial to environmental protection and cost reduction, expanding its potential application in the field of building insulation.

## 2. Materials and Methods

### 2.1. Materials

**Figure 1:**
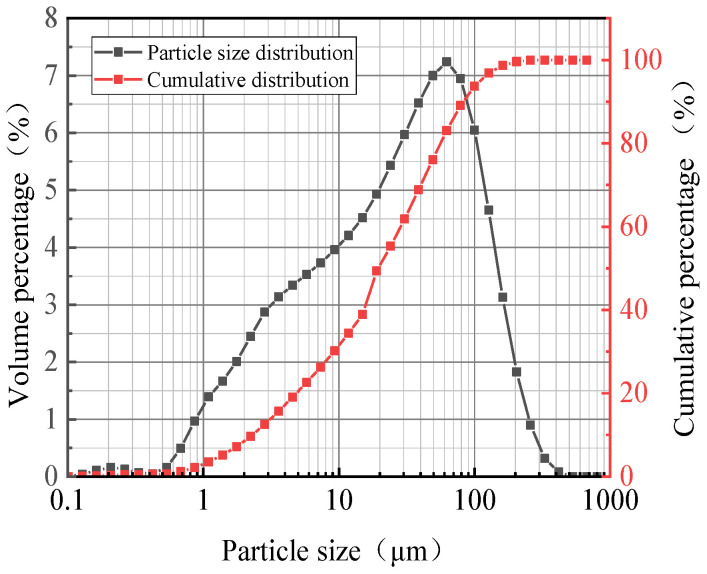
Particle size distribution of RCPs.

**Figure 2:**
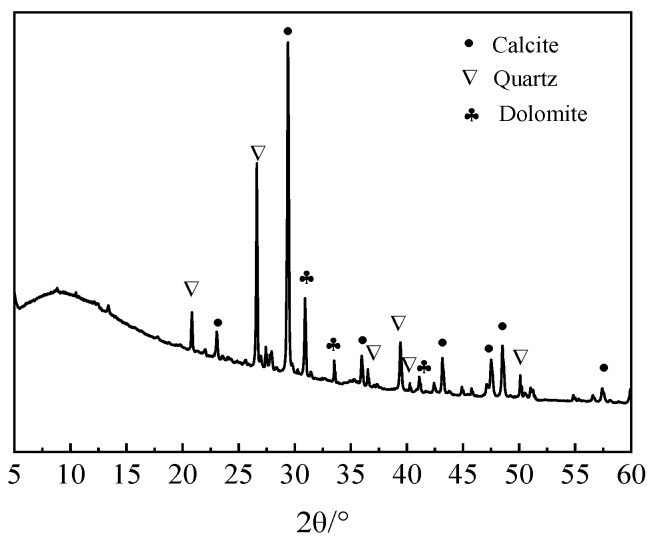
XRD pattern of RCPs.

The raw materials included recycled concrete powders (RCPs), slag, and fly ash. After 15 min of mechanical grinding, the specific surface area and average particle size of the RCPs were 329 m^2^/kg and 33.03 μm, respectively. The grade of the slag powder was S95, which was provided by Lanke Environmental Water Purification Materials Co., Ltd. (Gongyi, China). The fly ash was second-grade ash, which was provided by Yangzhou Huayun New Material Technology Co., Ltd. (Yangzhou, China). The chemical constitution of the RCPs, slag, and fly ash are shown in Table 1. Figure 1 and Figure 2 show the particle size distribution and the XRD pattern of RCPs, respectively.

The industrial water glass, with a modulus of 3.3, a solid content of 35.8%, and the appearance of transparent viscous liquid, was produced by Jiashan Yourui Refractory Materials Co., Ltd. (Jiangxing, China). Analytical purity sodium hydroxide with a sodium hydroxide content ≥ 96% was produced by Tianjin Kemio Chemical Reagent Co., Ltd. (Tianjin, China). The alkali activator was comprised of sodium hydroxide and water glass; the Na_2_O concentration was 17.1%, and the modulus of water glass was 1.4. The foaming agent used in this paper was analytically pure sodium dodecyl sulfate (K12), and the stabilizer was analytically pure calcium stearate.

### 2.2. Mix Proportions

ARCP-FC was prepared with a different W/C ratio, Na_2_O content, and foam content, accompanied by an RCPs:slag powder ratio of 50%:50%. The W/C ratios were 0.4, 0.45, and 0.5, and the Na_2_O contents were 6%, 8%, and 10%, respectively. The foam contents were 5%, 3.4%, and 1.8% in order to prepare the samples with apparent densities of 600 kg/m^3^, 800 kg/m^3^, and 1000 kg/m^3^, which were marked as A06, A08, and A10, respectively. The mix proportions of ARCP-FC with different W/C ratios are shown in Table 2. The mix proportions of ARCP-FC with different Na_2_O contents are shown in Table 3. The mix proportions of ARCP-FC with different mineral admixtures are shown in Table 4. The proportions of RCPS, slag, and fly ash were 6:4:0, 6:3:1, 6:2:2, 5:5:0, 5:4:1, and 5:3:2, with apparent densities of 600 kg/m^3^ and 800 kg/m^3^, respectively.

### 2.3. Sample Preparation

**Figure 3:**
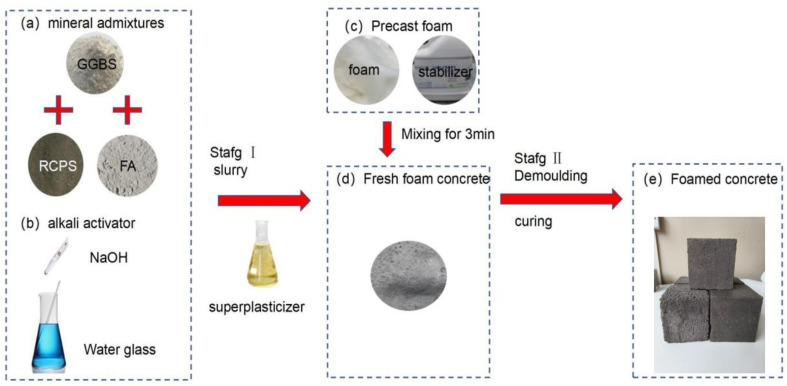
Preparation process of ARCP-FC.

Mixtures were prepared by: (1) mixing the RCPs, slag, and fly ash powder evenly and stirring slowly for 2 min; (2) adding the alkali activator and stirring with fresh water to prepare the slurry; (3) diluting the foaming agent with water to generate the foaming liquid and using the foaming machine to prepare the dense and stable foam; and (4) adding different foam ratios in ARCP-FC and mixing slowly for 1 min. The preparation process is illustrated in Figure 3.

### 2.4. Compressive Strength Test

Cube specimens of 100 mm^3^ of ARCP-FC were mixed for mechanical strength testing according to JG/T 266-2011 [29]. The curing temperature and relative humidity were 20 ± 1 °C and >90%, respectively. The pastes were dried to a fixed weight in the oven at 60 ± 5 °C at a curing age of 28 days.

### 2.5. Dry Apparent Density Test

The cube samples used for the dry apparent density were prepared according to GB/T 5486-2008 [30], and the pastes were dried to the fixed weight in the oven at 60 ± 5 °C for 28 days. The dry apparent density was obtained by the measurement of the mass and volume.

### 2.6. Water Absorption Test

**Figure 4:**
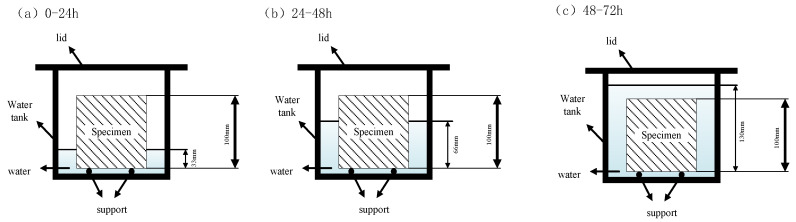
Schematic diagram of the water absorption measurement.

The cube specimens were prepared for the water absorption rate according to JG/T 266-2011. The steps comprised the following: (1) the initial mass (m0) of the samples before being soaked was weighed; (2) 1/3 of the height of the specimens was submerged underwater for the first 24 h at 20 ± 5 °C; water was then added to 2/3 of the height of the specimens and submerged for 24~48 h, and the specimens were soaked at 30 mm below the water level during 48~72 h, as shown in Figure 4; and (3) the specimens were then wiped with a wet cloth, and the quality (mg) of the specimens was measured. The water absorption is outlined in Equation (1).

(1) $$W_{R}=\frac{m_{g}-m_{0}}{m_{0}}\times 100\%$$

where: W_R_ is the water absorption (%), *m*_0_ is the mass of cube specimens before soaking (g); and *m*_g_ is the mass of cube specimens after soaking (g).

### 2.7. Porosity

**Figure 5:**
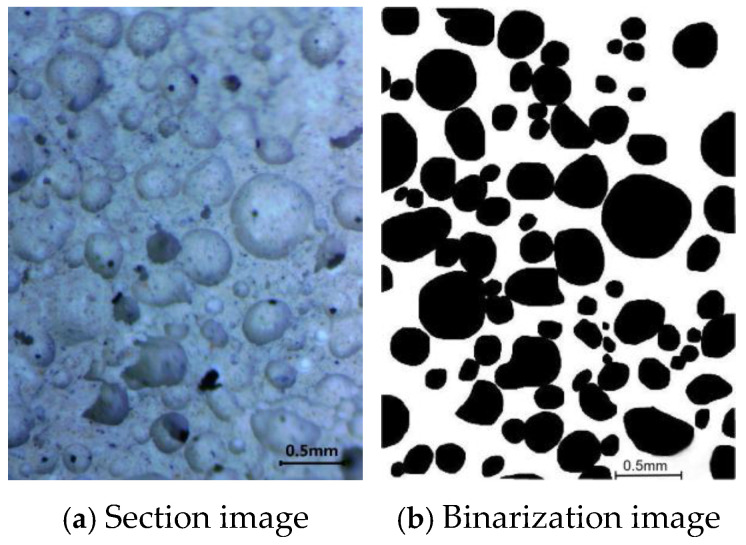
Sectional images of the air hole.

The pore structure was analyzed using the image analysis algorithm at a magnification of 7~45 times, and the porosity was studied using Image-Pro Plus using a German LIOO SZ745T microscope (LIOO, Attendorn, Germany). Section images were processed through binarization to obtain the required pore structural parameters; the binary processing results are shown in Figure 5. The average pore diameter is the average of the internal pore diameter of foamed concrete, which is usually calculated using the image analysis method. The hole shape factor refers to the degree to which the geometry of the pores deviates from the sphere. The hole shape factor S can be calculated according to Equation (2). The aperture is spherical when S = 1; the larger the S value, the more the aperture shape deviates from the sphere.

(2) $$S=\frac{L^{2}}{4\pi A}$$

where S is the pore shape factor, *L* is the perimeter (m), and *A* is the area of pore (mm^2^).

### 2.8. Thermal Conductivity Test

The cube bricks of 40 mm^3^ of ARCP-FC were mixed for thermal conductivity testing. The pastes were dried to a fixed weight at 60 ± 5 °C and were tested using a Hot Disk TPS 2500 (Hot Disk, Gothenburg, Sweden). The thermal conductivity was evaluated using the transient plane heat source. Thermal conductivity was surveyed using the transient plane heat source method. The test ranged from 0.005 to 500 W/(m·K), and the test temperature ranged from −40 °C to 600 °C.

### 2.9. Drying Shrinkage

According to the standard JC/T 603-2004 [31], specimens of 40 mm × 40 mm × 160 mm were evaluated for a drying shrinkage of ARCP-FC. The pastes were cured at 20 ± 1 °C and 50 ± 4% RH in the shrinkage curing box. The drying shrinkages at 3 d, 7 d, 14 d, 28 d, and 56 d were tested, respectively.

### 2.10. Micro-Structure Test

X-ray diffraction (D8 ADVANCE, Bruker, Billerica, MA, USA) was employed to discover the composition of hydrates. The crushed cement pieces were soaked in anhydrous ethanol to prevent further hydration at specified ages and dried at 60 °C. After being ground and screened with a 0.08 mm sieve, TG analysis was employed in a range of 0~1000 °C using a Pyris 1 TGA (PerkinElmer, Hopkinton, MA, USA). The field emission SEM was tested using a Hitachi-4800 (Hitachi, Tokyo, Japan).

## 3. Results

### 3.1. The Effect of W/C Ratio on the Performance of ARCP-FC

#### 3.1.1. Compressive Strength and Dry Apparent Density

**Figure 6:**
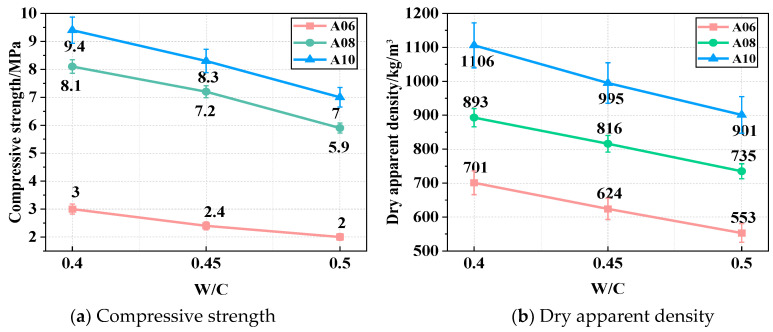
Compressive strength and dry apparent density of ARCP-FC with different W/C ratios.

Figure 6a shows the CS of ARCP-FC with W/C ratios of 0.4, 0.45, and 0.5. The CS of ARCP-FC decreased gradually as the W/C ratio increased. When the W/C ratio was 0.4, the CS of foamed concrete A06, A08, and A10 were 3.0 MPa, 8.1 MPa, and 9.4 MPa, respectively. As the W/C ratio increased to 0.45, the CS of foamed concretes A06, A08, and A10 decreased by 19.3%, 11.6%, and 8.1%, but decreased by 35.2%, 31.2%, and 25.0% as the W/C ratio was further increased to 0.5, respectively. The reason for this is that the consistency of slurry was reduced under the low W/C ratio, leading to the deformation and rupture of bubbles in foamed concrete. Figure 6b shows the dry apparent density of ARCP-FC at W/C ratios of 0.4, 0.45, and 0.5. The dry apparent density decreased with the increase in the W/C ratio. The densities of foamed concretes A06, A08, and A10 were 701, 893, and 1106 kg/m^3^ when the W/C ratio was 0.4, which increased by 16.8%, 11.6%, and 10.6% over the standard values of 600, 800, and 1000 kg/m^3^, according to JG/T 266-2011. When the W/C ratio was 0.45, the densities of foamed concretes A06, A08, and A10 were 624, 816, and 995 kg/m^3^, and they met the requirement of JG/T 266-2011. When the W/C ratio was 0.5, the densities were lower by 7.8%, 8.1%, and 9.9% than the value of JG/T 266-2011. However, the low consistency of slurry caused the float of bubbles on the surface and the non-uniform pore structure due to buoyant force. The bubbles in foamed concrete played a “dispersion-lubrication” role [32], which was similar to the role of a water reducer. The increase in the W/C ratio reduces the number of macropores and increases the number of micropores in the slurry. It is beneficial for the uniformity of internal pores, but it is not conducive to strength growth. Thus, the porosity of the structure increased, and the dry apparent density decreased with the increase in W/C ratios.

#### 3.1.2. Water Absorption

**Figure 7:**
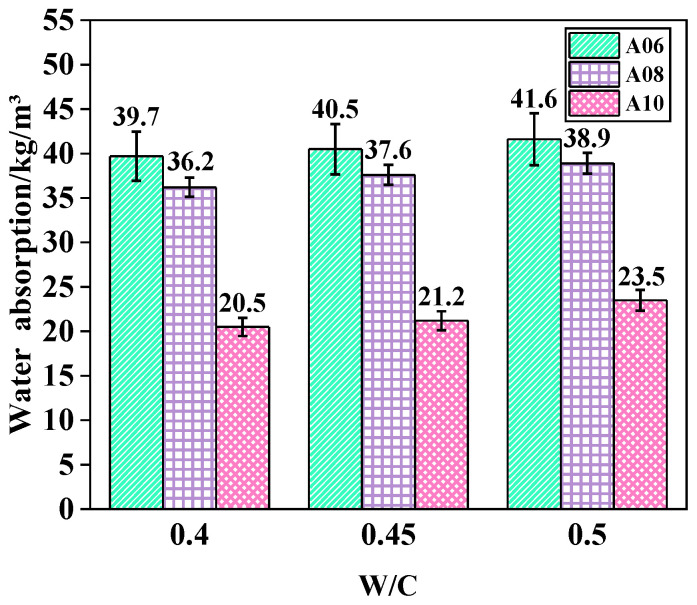
Water absorption of ARCP-FC with different W/C ratios.

Figure 7 reports the effect of W/C ratios on the water absorption of ARCP-FC; the W/C ratios were 0.4, 0.45, and 0.5, respectively. It indicates that the water absorption of foam concrete gradually increased under the same dry density as the water-cement ratio. The W50 met the requirements of the water absorption rate, which was lower than 50%, according to JG/T 266-2011. The “bleeding phenomenon” was observed in the slurry, and water was floated on the slurry surface. This was due to the fact that too much free water was evaporated to form more connected pore structure, thereby increasing the internal porosity and water absorption rate of ARCP-FC. However, there were fewer capillary pores and less growth of the water absorption due to the quick setting of ARCP-FC in the alkali activation system. Therefore, the water absorption rate of ARCP-FC increased with the decrease in dry density, and the optimal W/C ratio was determined to be 0.45. In conclusion, the hardened foamed concrete showed high CS, normal dry apparent density, and suitable water absorption.

### 3.2. Effect of Na_2_O Content on the Performance of ARCP-FC

#### 3.2.1. Compressive Strength and Dry Apparent Density

**Figure 8:**
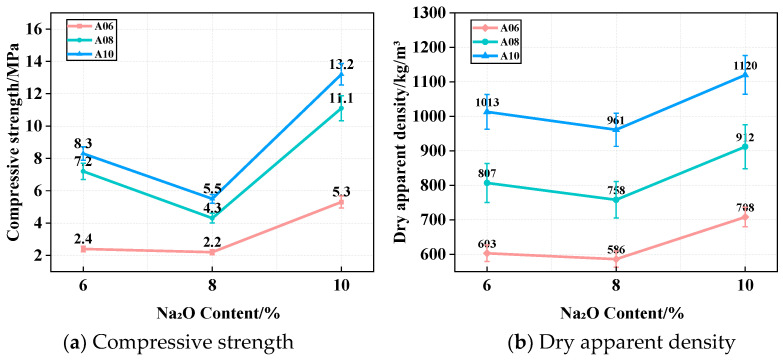
Compressive strength and dry apparent density of ARCP-FC with different Na_2_O contents.

An optimal W/C ratio of 0.45 was applied to prepare ARCP-FC. Figure 8a shows the CS of ARCP-FC with Na_2_O contents of 6%, 8%, and 10%. The CS of ARCP-FC decreased first and then improved with the increase in Na_2_O dosage. The CS of foamed concretes A06, A08, and A10 were 2.4 MPa, 7.2 MPa, and 8.3 MPa, respectively. However, it decreased by 8.3%, 40.2%, and 33.7% when the Na_2_O content was 8%. Furthermore, when the Na_2_O content reached 10%, the strength of the foamed concrete increased by over 50%, reaching 5.3, 11.1, and 13.2 MPa. The reason for this is that the OH^−^ concentration in the system improved with the increase in Na_2_O dosage, leading to more agglomerate hydrates and the decreased strength of ARCP-FC. When the Na_2_O content reached 10%, high Al and Si contents resulted in the enhanced dissolution of precursors and the acceleration of the hydration reaction [33,34]. Moreover, the increase in Na^+^ content accelerated the decomposition of reactants and activated their activity, generating more (C, N)-A-S-H gel and improving CS significantly [35,36].

Figure 8b shows the dry apparent density of ARCP-FC with Na_2_O contents of 6%, 8%, and 10%. The dry apparent densities of foamed concretes A06, A08, and A10 were 603, 807, and 1013 kg/m^3^, and they met the requirement of JG/T 266-2011. The rupture of foam occurred in the process during hardening, and the rapid hardening retained more foam, reducing the dry apparent density of ARCP-FC. The setting time was gradually shortened with the increase in Na_2_O content. However, foam stability decreased and the breakage risk increased due to the high consistency of the slurry when the Na_2_O content was excessive.

#### 3.2.2. Water Absorption

**Figure 9:**
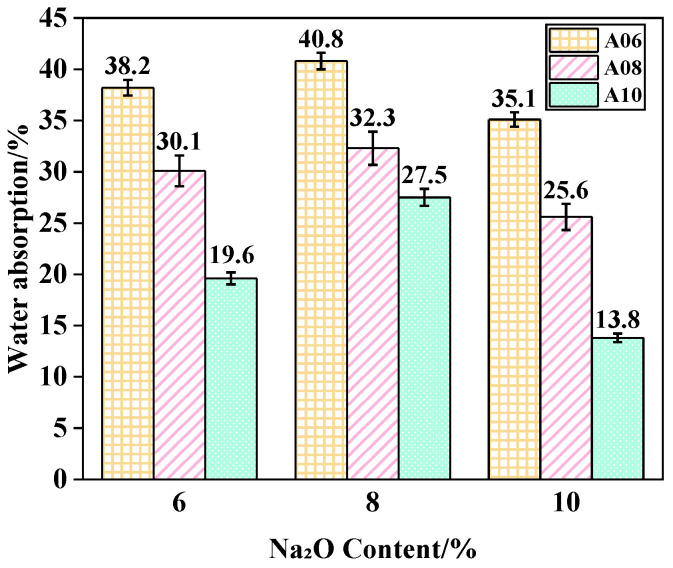
Water absorption of ARCP-FC with different Na_2_O contents.

Figure 9 illustrates the effect of Na_2_O content on the water absorption of ARCP-FC; the Na_2_O contents were 6%, 8%, and 10%, respectively. The results indicate that the water absorption rate initially rised and then falled with the Na_2_O content growth. After 6% Na_2_O was added, the water absorption of foamed concretes A06, A08, and A10 was 38.2%, 30.1%, and 19.6%, and reached 40.8%, 32.3%, and 27.5% with an Na_2_O content of 8%, respectively. The water absorption of ARCP-FC met the requirements of JG/T 266-2011, in which it needed to be lower than W50 (lower than 50%). The increase in water absorption was because of the increasing Na_2_O content; the Na_2_O content and the alkali content were positively correlated. However, the excessive Na^+^ ions that remained after balancing the [SiO_4_]^4−^ and [AlO_4_]^5−^ tetrahedrons reacted with CO_2_, and thus led to carbonization [37]. Therefore, the inhomogeneity and defect of the structure of ARCP-FC increased, increasing the water absorption. The excessive Na_2_O content of 10% showed an adverse effect on the porosity and water absorption of ARCP-FC.

#### 3.2.3. Pore Structures

**Figure 10:**
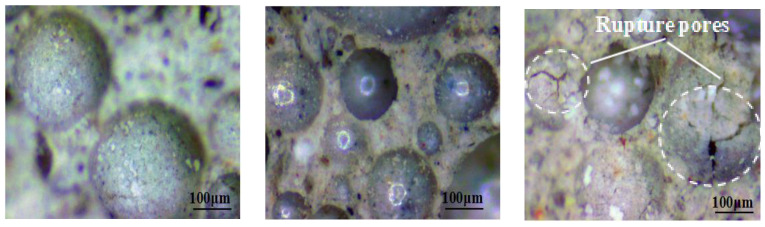
Pore structure of the ARCP-FC with different Na_2_O contents.

The pore structures of ARCP-FC are shown in Figure 10. The Na_2_O contents were 6%, 8%, and 10%, respectively. The porosity, with different Na_2_O additions, increased by 45.3%, 41.2%, and 38.9%, respectively. Moreover, 10% Na_2_O content significantly increased the alkali amount but reduced the foam stability. The pore size was uniform and round when the Na_2_O content was 6%. The top surface was pitted and flat when the Na_2_O content was 8%. However, the pore was broken on the top when the Na_2_O content was 10%. Meanwhile, the foam cured more rapidly, and the porosity was higher. It revealed comparable pore diameter ranges (100–500 μm) between the developed ARCP-FC. The conventional formulations observed in previous studies [17,18,20] are similar.

### 3.3. Effect of Mineral Admixtures on the Behavior of ARCP-FC

#### 3.3.1. Compressive Strength and Dry Apparent Density

**Figure 11:**
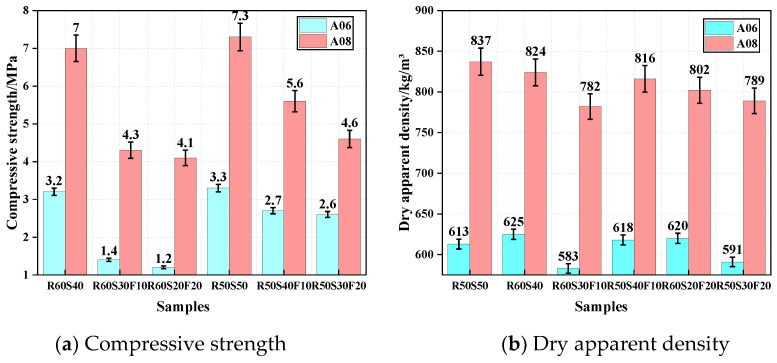
Compressive strength and dry apparent density of ARCP-FC with different mineral admixtures.

Figure 11a reports the CS of ARCP-FC with different mineral admixtures. The CS of foamed concrete increased with the increase in slag content. For the CS of R60S40, A06 and A08 were 3.2 MPa and 7.0 MPa. For the sample R50S50, the CS of A06 and A08 increased to 3.3 and 7.3 MPa. With the increase in fly ash content, the CS of the foamed concrete decreased. For the sample R60S30F10, the CS of A06 and A08 were reduced by 54.2% and 39.2%. However, the CS of R60S20F20 of A06 and A08 was reduced by 63.7% and 40.7%, respectively. Therefore, the higher slag content resulted in the higher strength of foamed concrete. Figure 11b shows the dry apparent density of ARCP-FC with different mineral admixtures. The change in fly ash content showed little impact on the dry apparent density. The dry apparent density of A06 and A08 ranged within 583~625 and 782~837 kg/m^3^, respectively. The addition of slag powder led to more closed pores and a denser structure.

#### 3.3.2. Water Absorption

**Figure 12:**
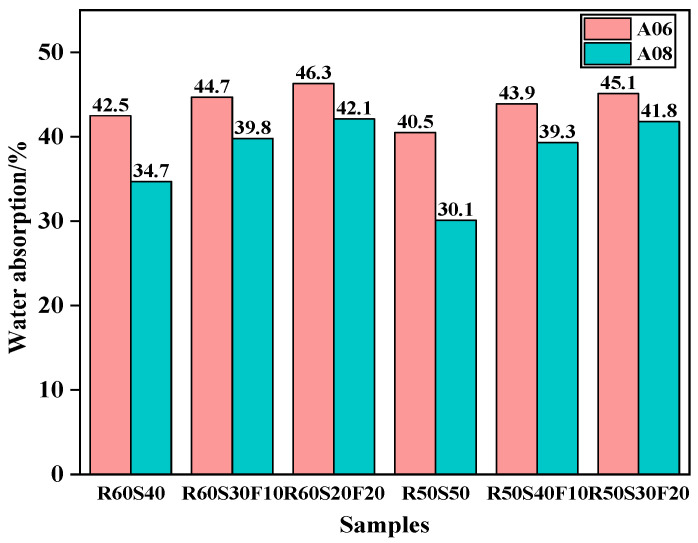
Water absorption of ARCP-FC with different mineral admixtures.

Figure 12 reports the water absorption of ARCP-FC with different mineral admixtures. The water absorption of foamed concrete gradually enhanced with the increase in fly ash content. For sample R60S20F20, the water absorption of A06 and A08 reached the highest values of 46.3% and 42.1% and met the requirement of being lower than 50%. Comparative analysis reveals a 12.5% increase in water absorption of A06 when comparing the R60S20F20 containing fly ash with R50S50. This performance variation can be attributed to the synergistic pozzolanic reactions between constituents, which refine the pore structure through enhanced hydration product formation while simultaneously increasing capillary pore connectivity. The incorporation of slag induces a significant 13.3% reduction in the water absorption of A08, correlating with enhanced structural integrity through accelerated matrix densification. The reason for this is that fly ash showed limited pozzolanic activity in the short term, leading to the loose structure with more bubbles, along with increased porosity and water absorption. Conversely, the incorporation of slag powder promotes secondary C(N)-A-S-H gel formation, effectively refining capillary pore networks while reducing total porosity, thereby decreasing the water absorption of ARCP-FC significantly.

### 3.4. Pore Structure and Thermal Conductivity

#### 3.4.1. Pore Size Distribution

**Figure 13:**
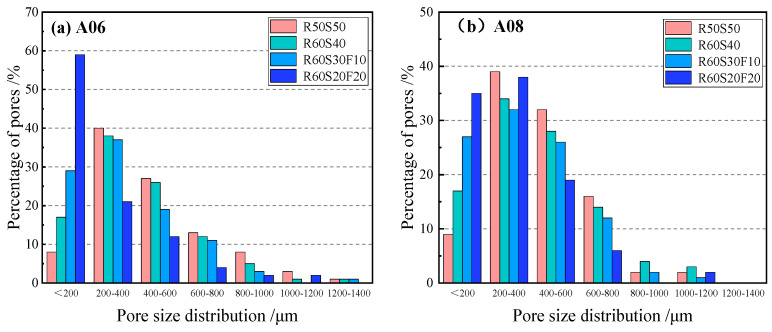
Pore size distribution of ARCP-FC.

Figure 13 reports the pore size distribution of ARCP-FC with mineral admixtures. The pore size of foamed concrete with the fly ash was mainly distributed in 0~200 μm and 200~400 μm. For sample R60S30F10, the proportion of A06 of 0~200 μm was 29%, and that of 200~400 μm was 37%. For sample R60S20F20, the proportion of A06 of 0~200 μm was 59%, and that of 200~400 μm was 21%. The small pores of ARCP-FC were improved as the fly ash ratio increased. Because of the “morphological effect” of fly ash, the fluidity of the slurry was improved while the viscosity was reduced, lowering the extrusion damage to the foam. At the same time, fly ash played the role in the homogenization and allowed the bubbles to be evenly distributed, increasing the proportion of small pores.

#### 3.4.2. Average Pore Diameter and Pore Shape Factor

**Figure 14:**
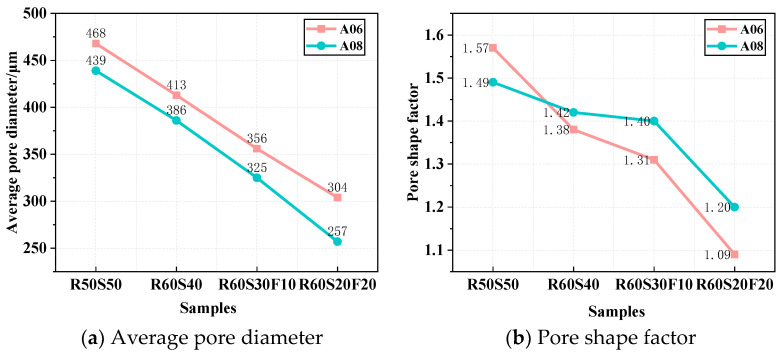
Average pore diameter of ARCP-FC.

The average pore diameter can accurately reflect the effects of admixtures on the pore structure of foamed concrete. Figure 14a shows the average pore diameter of ARCP-FC. It gradually decreased with the increasing fly ash ratio, and A06 and A08 were reduced from 468 to 304 μm and from 439 to 257 μm, respectively. The reduced average pore size is related to the “ball bearing role” of glass beads in fly ash, lowering the water demand and surface tension of foamed concrete. Figure 14b shows the pore shape factor of ARCP-FC with different mineral admixtures. It gradually reduced with the increasing fly ash ratio. When the pore shape factor was closer to 1.0, the shape of pores was closer to being round. When the slag powder content was too high, the pore shape factor increased, which was not conducive to the thermal insulation performance. The pore shape factors of A06 and A08 showed little difference. The pore shape factor R60S20F20 of A06 reached 1.09, indicating the excellent pore structure. The deformation degree of pores was improved with the rounded shape due to the “micro filling effect” of fly ash. Another reason for this is that the density of fly ash is low [38]. The incorporation of fly ash improved the pore size distribution and pore shape factor of foamed concrete.

#### 3.4.3. Thermal Conductivity

**Figure 15:**
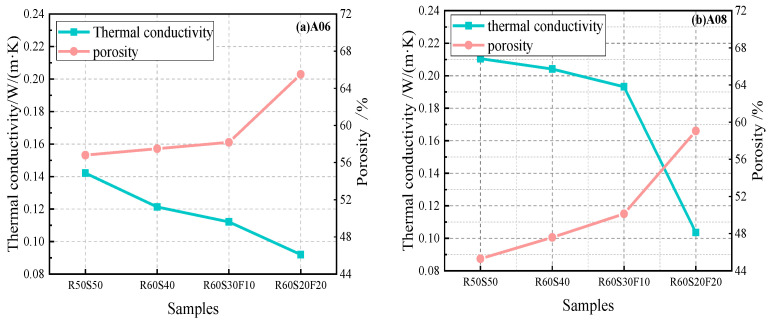
Correlation of thermal conductivity and porosity of ARCP-FC.

Figure 15 reports the relationship of thermal conductivity and porosity. For sample R60S20F20, the porosity of A06 increased from 56.8% to 65.5%, and the thermal conductivity decreased from 0.1421 to 0.0920 W/(m·K) with the increase in fly ash content. The porosity of A08 increased from 45.3% to 59.1%, and the thermal conductivity decreased from 0.2105 to 0.1036 W/(m·K). The lower density resulted in greater porosity and less thermal conductivity. In conclusion, the porosity and thermal insulation performance of foamed concrete increased while the thermal conductivity reduced with the increasing fly ash ratio.

**Figure 16:**
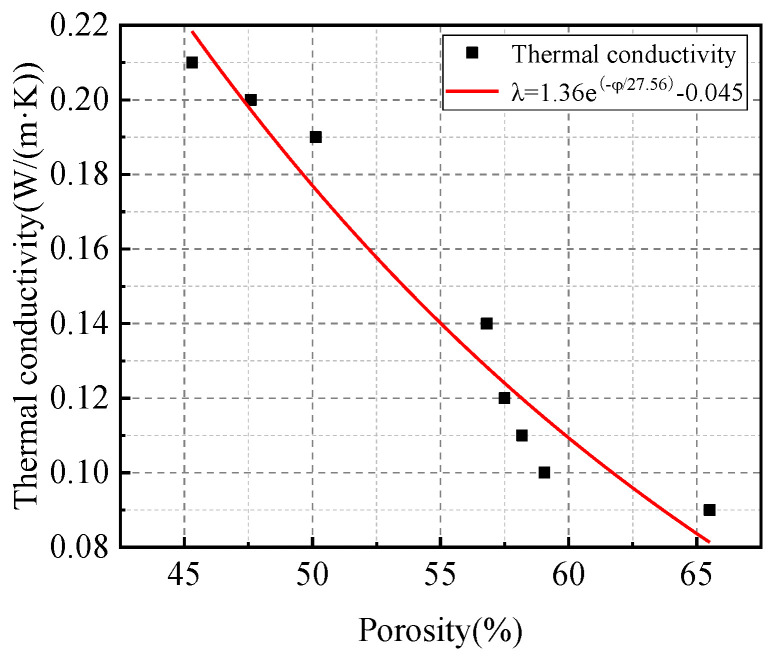
Fitting curve of the relationship between porosity and thermal conductivity.

Figure 16 shows the exponential fitting relationship of thermal conductivity and porosity. The thermal conductivity decreased with the increase in porosity. The fitting curve is expressed by Equation (3). There were positive relationships between the thermal conductivity and porosity when the porosity increased from 45% to 65%, and the thermal conductivity approached 0 as the porosity approached 100%. This is because the thermal conductivity of the bubble structure was lower than that of the solid. The fitting curve of thermal conductivity and porosity is shown in Equation (3).

(3) $$\lambda =1.36e^{\left(-\varphi /27.56\right)}-0.045$$

where λ is the thermal conductivity, W/(m·K); φ is porosity, %.

#### 3.4.4. Drying Shrinkage

**Figure 17:**
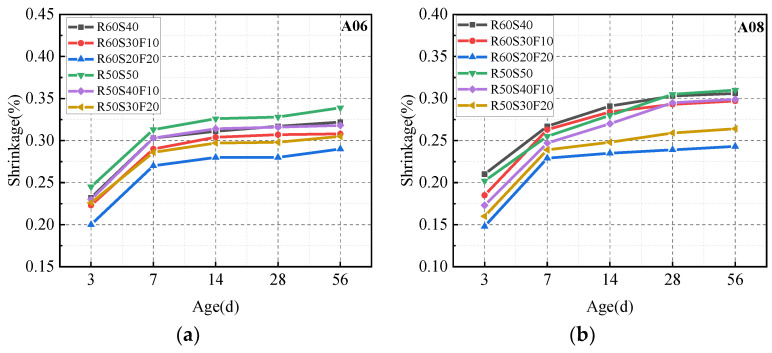
Dry shrinkage of ARCP-FC.

Figure 17 shows the drying shrinkage of ARCP-FC under different mineral admixtures. The drying shrinkage increased with the extension of drying time. The dry shrinkage increased significantly at 7 days and stabilized after 28 days and then declined with the increase in the fly ash dosage. When there was no fly ash added (R50S50), the 56-day dry shrinkage of A06 and A08 of ARCP-FC showed the highest values of 0.322% and 0.310%, respectively. After 20% fly ash was added (R60S20F20), the 56-day dry shrinkage of A06 and A08 of ARCP-FC were 0.293% and 0.243%, respectively, which were 9.0% and 21.6% lower than R50S50. This is because fly ash partially replaced slag powder, and the activity of fly ash was lower than that of the slag powder in the early stage, resulting in fewer hydrates and smaller shrinkage. Therefore, fly ash can reduce the shrinkage of ARCP-FC. In addition, the drying shrinkage of A08 was smaller than that of A06.

### 3.5. Micro-Structure

#### 3.5.1. XRD

**Figure 18:**
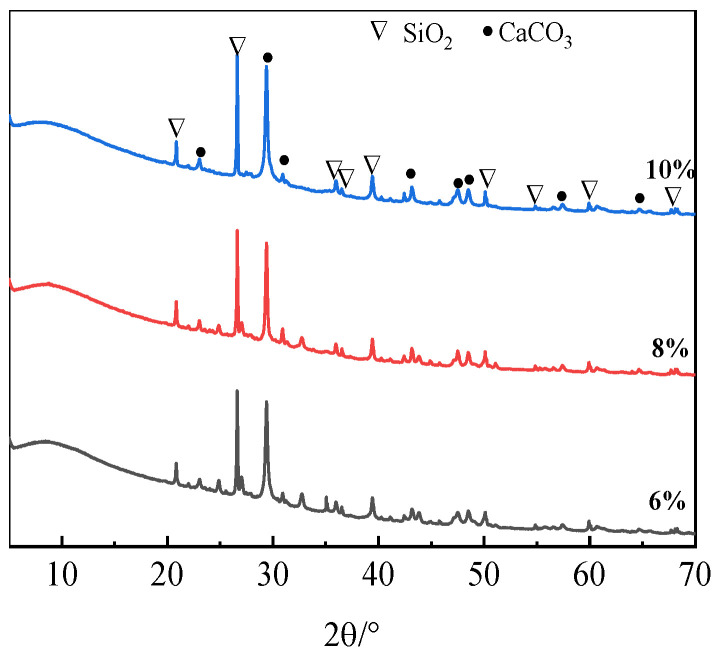
XRD spectrum of ARCP-FC with different Na_2_O contents.

The XRD results of ARCP-FC with different Na_2_O additions of 6%, 8%, and 10% are shown in Figure 18. It indicates that there were no new hydration products after the incorporation of RCPs. The diffraction peaks of the silica and calcite phases remained the same, and the main phases of ARCP-FC were calcite (d = 0.3859 nm, 0.3034 nm), quartz (d = 0.4255 nm, 0.3342 nm), and amorphous phases. The source of the strength of ARCP-FC was carbonation and hydration products. CaCO_3_ originated from the C-S-H gel and the carbonization of Ca(OH)_2_, and the amorphous phase of aluminosilicate gel was attributed to the alkali activation of RCPs and slag. The reaction processes were as follows: the contents of Na^+^, OH^−^ ions were enhanced with the increase in the Na_2_O ratio and generated more (C, N)-A-S-H gel. (C, N)-A-S-H gel was also generated by the double activation of “depolymerization-condensation” when the concentration of the [SiO_4_]^4−^ group increased to the critical concentration [39].

#### 3.5.2. TG

**Figure 19:**
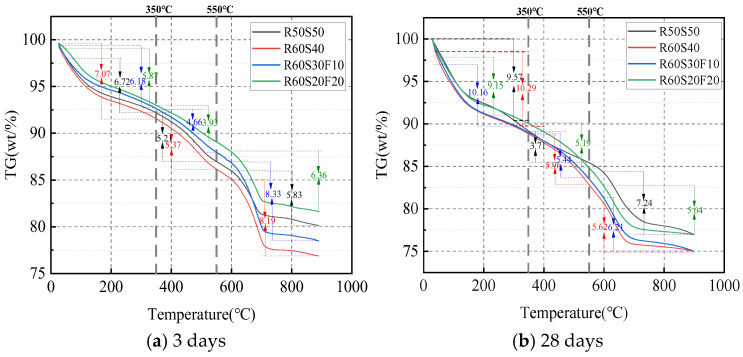
TG curves of ARCP-FC.

Figure 19 shows the TG results of ARCP-FC at 3 days and 28 days. The quality loss was divided into three stages of 20 °C~300 °C, 350 °C~ 550 °C, and 600 °C~900 °C. The mass loss increased with the rising slag powder during 20 °C~300 °C. This is due to the high activity of slag powder producing more hydrates of C(N)-A-S-H gel. The weight loss was relatively small during 350 °C~550 °C. It was inferred that a small quantity of Ca(OH)_2_ decomposed. However, Ca(OH)_2_ was easily carbonized into CaCO_3_, due to the secondary hydration between Ca(OH)_2_ with the active admixtures. Further investigation is needed to determine whether Ca(OH)_2_ was generated. The sample exhibited significant weight loss due to the thermal decomposition of CaCO_3_ during 600 °C~900 °C, and the weight loss enlarged with the increase in RCPs content. This is because the RCPs contained a large amount of CaCO_3_, which was decomposed to CO_2_ and CaO, while a small amount of dolomite decomposed to CO_2_, MgO, and CaO. The amount of hydration products generated by ARCP-FC under different mineral admixtures was as follows: R60S40 > R60S30F10 > R50S50 > R60S20F20.

#### 3.5.3. SEM

**Figure 20:**
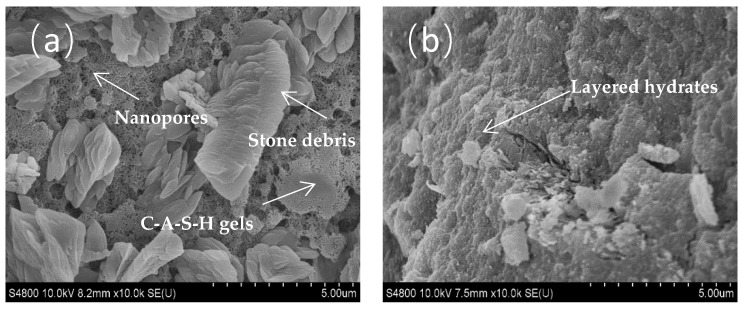
SEM images of ARCP-FC at different ages. (**a**) 3 days; (**b**) 28 days.

Figure 20 shows the SEM images of ARCP-FC at 3 days and 28 days. Some microstone debris did not participate in the alkali-activated reaction. Although denser pores appeared in pastes, limited polymerization and weakly connected (C, N)-A-S-H gel led to the low early strength and high porosity of ARCP-FC at 3 days, as shown in Figure 20a. As the hydration proceeded, pastes showed a denser microstructure, and the strength was significantly improved. It can be seen that the internal pores were uniform and dense. Figure 20b shows the uniform and denser structure of ARCP-FC at 28 days. The nanopores disappeared, and the microstone debris, along with (C, N)-A-S-H gel, made up the denser hardened paste due to the alkali activator. Foamed concrete prepared with alkali-activated RCPs showed a denser pore structure and promising thermal insulation properties.

## 4. Discussion

### 4.1. Alkali Activation Improves the Thickness of the Pore Wall

A certain number of studies in the literature indicate that [40,41] the thickness and strength of the pore wall structure are improved with the increase in Na_2_O content, but an excessive dosage of Na_2_O causes cracks on the surface of the foam, forming a stress concentration of pores on the round shape under the pressure of wall thickness. Ultimately, it is unable to resist self-weight, the pore starts to crack from the top, and the crack spreads. In summary, the hydrolysis process of water glass not only generates OH^−^ ions but also generates [SiO_4_]^4−^ groups to promote the reaction of “depolymerization-polycondensation”, which has a dual activation effect. However, in the preparation of ARCP-FC, it also has a limited dosage. Otherwise, it is easy to crack the pore wall, affecting the strength and thermal insulation of ARCP-FC.

### 4.2. Fly Ash Improves Thermal Performance

Fly ash has the function of improving the pore structure of hardened paste; the increase in fly ash content reduces the number of macropores and increases the number of micropores in the slurry and reduces the thermal conductivity. The thermal performance of foamed concrete can be improved by reasonable composition of recycled concrete powders, slag, and fly ash.

### 4.3. Applications Prospect

ARCP-FC has the characteristics of being low cost (without cement), having a short setting time, good thermal insulation performance, etc. It can also be used as external insulation boards or insulation layers for new buildings and solve the contradiction between compressive strength and dry apparent density. However, the alkali-activated binder system containing slag demonstrates elevated cracking propensity [42,43]. The incongruent dissolution rates between amorphous/crystalline phases in ARCP-FC create shrinkage-induced cracks.

## 5. Conclusions

Recycled concrete powders, slag powder, water glass, foam stabilizer, and foaming agent were applied to prepare ARCP-FC with densities of 600, 800, and 1000 kg/m^3^. The mix proportion, preparation, and properties of ARCP-FC were analyzed. The conclusions are summarized as follows:

(1) Slag powder improved early strength but has an adverse effect on the functionality of ARCP-FC. Fly ash improved the deformation, and the pore was closed to the sphere, reducing the shrinkage and thermal conductivity. The optimal mixture of ARCP-FC was R60S20F20, which consisted of 60% recycled concrete powders, 20% slag, and 20% fly ash.
(2) In the optimal mixture of ARCP-FC, the density, porosity, compressive strength, and thermal conductivity of ARCP-FC were 800 kg/m^3^, 59.1%, 4.1 MPa, and 0.1036 W/(m·K), respectively. ARCP-FC solved the contradiction between compressive strength and dry apparent density, making it a promising building material for external insulation boards and insulation layers.
(3) Fly ash improved the deformation and the pore was closed to the sphere, reducing the shrinkage and thermal conductivity. ARCP-FC solved the contradiction between compressive strength and dry apparent density, making it a promising building materials for external insulation boards and insulation layers.

## Figures and Tables

**Table 1:** Chemical constitution of raw materials (wt.%).

Material	SiO_2_	CaO	Al_2_O_3_	Fe_2_O_3_	MgO	K_2_O	TiO_2_	Na_2_O	Others
RCPs	34.3	42.1	8.2	6.2	4.5	1.2	1.0	1.0	1.5
Slag	30.0	38.1	13.6	0.6	12.5	0.4	0.6	0.3	3.9
Fly ash	61.9	2.4	28.8	2.5	0.8	1.5	1.04	0.3	0.76

**Table 2:** Mix proportions of ARCP-FC with different W/C ratios.

No.	Density (kg/m^3^)	RCPs (%)	Slag (%)	W/C Ratio	Na_2_O (%)	Foam Content (%)
FC1	600	50	50	0.4	6	5
FC2		50	50	0.45	6	
FC3		50	50	0.50	6	
FC4	800	50	50	0.4	6	3.4
FC5		50	50	0.45	6	
FC6		50	50	0.50	6	
FC7	1000	50	50	0.4	6	1.8
FC8		50	50	0.45	6	
FC9		50	50	0.50	6	

**Table 3:** Mix proportions of ARCP-FC with different Na_2_O contents.

No.	Density (kg/m^3^)	RCPs (%)	Slag (%)	W/C Ratio	Na_2_O (%)	Foam Content (%)
FCN1	600	50	50	0.45	6	5
FCN2		50	50	0.45	8	
FCN3		50	50	0.45	10	
FCN4	800	50	50	0.45	6	3.4
FCN5		50	50	0.45	8	
FCN6		50	50	0.45	10	
FCN7	1000	50	50	0.45	6	1.8
FCN8		50	50	0.45	8	
FCN9		50	50	0.45	10	

**Table 4:** Mix proportions of ARCP-FC with different mineral admixtures.

No.	RCPs (%)	Slag (%)	Fly Ash (%)	W/C Ratio	Na_2_O (%)
R60S40	60	40	0	0.45	6
R60S30F10	60	30	10	0.45	6
R60S20F20	60	20	20	0.45	6
R50S50	50	50	0	0.45	6
R50S40F10	50	40	10	0.45	6
R50S30F20	50	30	20	0.45	6

## Data Availability

The original contributions presented in the study are included in the article, further inquiries can be directed to the corresponding author.
